# Cecum perforation due to tuberculosis in a renal transplant recipient: a case report

**DOI:** 10.1186/1752-1947-3-132

**Published:** 2009-11-18

**Authors:** Sinan Carkman, Volkan Ozben, Erman Aytac

**Affiliations:** 1 Department of General Surgery, Istanbul University, Cerrahpasa Medical Faculty, Istanbul, Turkey

## Abstract

**Introduction:**

Tuberculosis can present in many varied clinical situations in immunosuppressed patients. It has been reported that the sigmoid colon is the most common site for colonic perforation in renal transplant recipients and diverticulitis is its most common cause. Cecal perforation because of tuberculosis is extremely rare in a renal transplant recipient. We present the case of a renal transplant patient with cecal perforation due to tuberculosis, 10 years after renal transplantation.

**Case presentation:**

A 39-year-old Caucasian man, who was a renal transplant recipient, was admitted to our emergency surgery unit with an acute abdomen. A cecal perforation was found at exploratory laparotomy, and a right hemicolectomy with an end ileostomy and transverse colonic mucous fistula were performed. Necrotizing granulomatous colitis due to tuberculosis was reported in the histopathologic examination.

**Conclusion:**

Colonic perforations in immunosuppressed patients may have unusual presentations and unusual causes. Tuberculosis infection should be considered in the differential diagnosis during the histopathologic evaluation in immunocompromised patients such as renal transplant recipients.

## Introduction

Patients with end-stage renal disease and renal transplant recipients suffer from tuberculosis (TB) more than other individuals because of their chronic immunosuppression. In transplant patients, the rate of active TB is 0.48% in the United States and 11.8% in India [1]. Diagnosis of TB is difficult and early diagnosis is essential as delay in the diagnosis and treatment results in progression of the disease with increased risk for renal damage and mortality [2]. Lethal complications in solid organ transplants notably include colonic complications, and colonic perforation has been of particular concern due to the high risk of mortality [3]. It has been reported that the sigmoid colon is the most common site for colonic perforation in renal transplant recipients and diverticulitis is the most common cause of perforation [4]. Cecum perforation due to TB is extremely rare. We present the case of a renal transplant patient with cecal perforation due to TB, 10 years after renal transplantation.

## Case presentation

A 39-year-old Caucasian man was admitted to our emergency surgery unit with complaints of acute abdominal pain, fever and chills. His past medical history revealed that 10 years previously, he had undergone right renal transplantation following 18 months of hemodialysis due to end stage renal failure secondary to amyloidosis. He had been treated with immunosuppressive therapy for nearly 10 years (prednisolone 500 mg, tacrolimus 1 mg, mycophenolate sodium 360 mg) after he developed membranous glomerulonephritic chronic renal rejection. He had been admitted to our chest medicine clinic 10 months before this admission with complaints of pleuritic chest pain, fever and weight loss. Radiological (chest X-ray, and chest computed tomography (CT)) and laboratory (blood culture, urinary antigen, tuberculin skin testing) tests for TB were inconclusive and he was diagnosed with pneumonia. His clinical condition gradually improved with antibiotic therapy (ceftriaxone 2 g/day). However, he sometimes suffered from night sweats and weight loss. His family history revealed that his father had been diagnosed with pulmonary tuberculosis 7 years earlier for which the father had received rifampicin.

**Figure 1:**
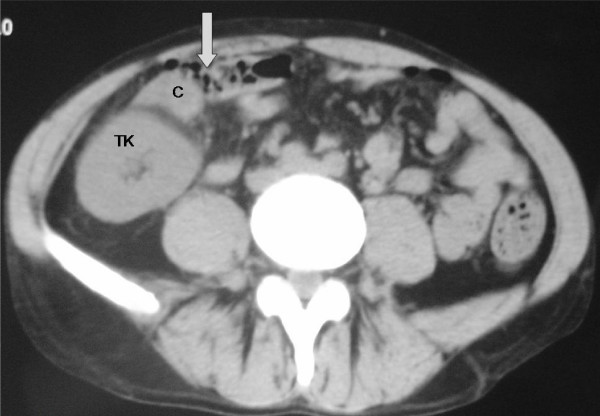
**Intra-abdominal free air identified on abdominal computed tomography scan**.

On physical examination, the patient appeared septic and unwell. His vital signs were: blood pressure: 130/80 mmHg, heart rate: 96 beats/minute, axillary temperature: 38.3°C, respiratory rate: 28/minute, oxygen saturation: 96%. Palpation of the abdomen revealed diffuse rigidity and rebound tenderness. Painful rectal ulcers were noted on digital rectal examination. All of the serologic and laboratory findings were normal except for white blood cell count (WBC): 14,900 cells/mm^3^, hematocrit (Hct): 28.7%, blood urea nitrogen (BUN): 78 mg/dl, creatinine: 3.1 mg/dl, C-reactive protein: 168 U/dl. Subdiaphragmatic free air was detected on plain chest X-ray study. An abdominal CT scan revealed pericecal inflammation and bowel perforation without any evidence of distal bowel obstruction (Figure 1).

**Figure 2:**
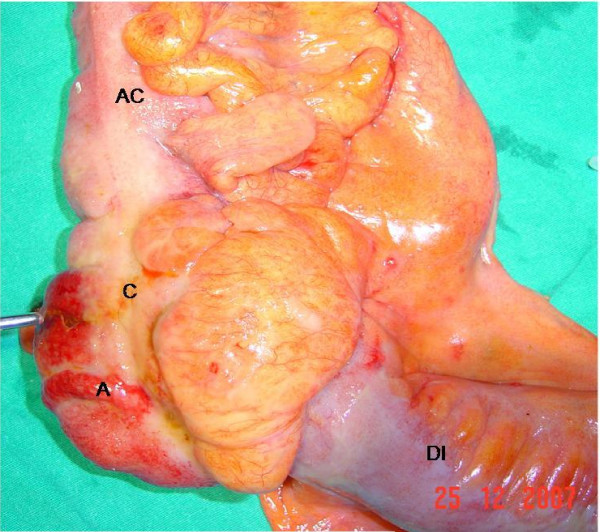
**Macroscopic view of the specimen**. The cecal perforation is shown with an instrument (AC: ascending colon, C: cecum, A: appendix, DI: distal ileum).

**Figure 3:**
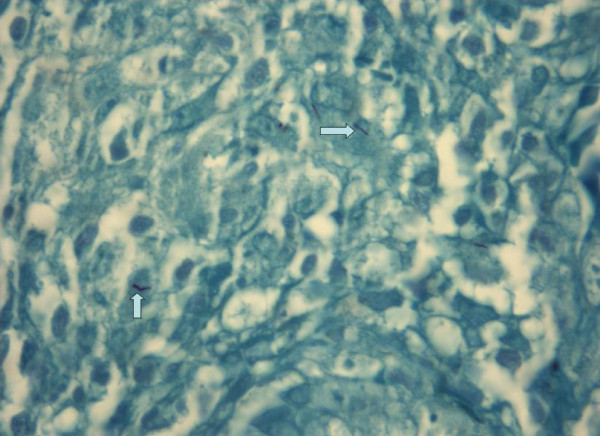
**Arrows indicate the tuberculosis bacilli in the colonic mucosa stained with Ziehl-Neelsen dye**.

An emergent exploratory laparotomy was performed and during exploration, intra-abdominal minimal purulent fluid, pseudomembranes, multiple lymphadenopathies measuring up to 1 cm in the mesenteric root and cecal perforation were detected (Figure 2). A right hemicolectomy with end ileostomy and transverse colonic mucous fistula was performed. Necrotizing granulomatous colitis due to TB was reported in the histopathologic examination (Figure 3). An antituberculosis drug regimen (pyrazinamide, rifampicin, isoniazid, ethambutol) was added to the patient's immunosuppressive treatment. The early postoperative period was uneventful and the patient improved rapidly. The rectal ulcers also regressed clinically. Ileostomy and colostomy closure were scheduled to be performed after full recovery.

## Discussion

The varied presentation of TB after transplantation is a challenge to the physician because immunosuppression masks the clinical course of TB and is difficult to interpret. Intestinal TB should be considered when a transplant recipient shows abdominal symptoms with no clear evidence of another infection [5-7]. Crohn's disease, amebiasis, carcinoma of the colon, *Yersinia *enterocolitis, gastrointestinal histoplasmosis, and peri-appendiceal abscesses closely simulate intestinal TB [8]. When TB is diagnosed during routine clinical follow-up, conventional antituberculosis agents are effective. However, when emergent clinical complications such as obstruction, perforation or fistula formations occur, surgery will be needed. Most patients with intestinal TB are diagnosed post mortem or after exploratory laparotomy and bowel resection. It has been reported that the sigmoid colon is the most common site for colonic perforation in renal transplant recipients and that diverticulitis is the most common cause of perforation [4]. Stelzner *et al*. reported a colonic perforation rate of 2.1% and a mortality rate of 38% in 1401 renal transplant recipients [9]. Patients with renal failure who are undergoing dialysis are at risk for colonic perforation due to chronic constipation, electrolyte imbalance, dehydration, inactivity and corticosteroid treatment, [4,10].

Our patient, who is a renal transplant recipient, presented with an acute abdomen. During abdominal exploration, a cecal perforation was diagnosed. A right hemicolectomy with an end ileostomy and transverse colonic mucous fistula was performed. We did not perform a primary intestinal anastomosis because there was diffuse peritonitis, and because, with the patient's immunosuppression, failure of the anastomosis was probable.

## Conclusion

The symptoms of TB are variable depending on the location of the disease and the immune condition of the patient. In our patient, a renal transplant recipient, TB caused cecal perforation. The location of the perforation site and the reason for the perforation were unusual for renal transplant recipients. An antituberculosis drug regimen was added to his treatment after the identification of TB infection by histopathology. The patient has improved rapidly with the antituberculosis therapy. TB infection should be considered in the differential diagnosis during the histopathologic evaluation of immunocompromised patients such as renal transplant recipients.

## Abbreviations

BUN: blood urea nitrogen; CRP: C-reactive protein; CT: computed tomography: Hct: hematocrit; TB: tuberculosis; WBC: white blood cell count.

## Consent

Written informed consent was obtained from the patient for publication of this case report and any accompanying images. A copy of the written consent is available for review by the Editor-in-Chief of this journal.

## Competing interests

The authors declare that they have no competing interests.

## Authors' contributions

SC and VO were the surgeons who performed the operation and close follow-up of the patient. VO and EA analyzed and interpreted the patient data regarding transplant and VO was the major contributor in writing the manuscript. All authors read and approved the final manuscript.
